# Supplementary material for: Evaluating the Impact of Regulatory Guidelines on Market Adoption and Implementation of Telehealth for COPD Patients: A Systematic Literature Review
Source: Healthcare (Basel). 2025 Nov 11;13(22):2858. doi: 10.3390/healthcare13222858 (PMC12652534; doi:10.3390/healthcare13222858)
Supplement: Supplementary file 1 [file healthcare-13-02858-s001.zip › Supplementary Table S1.pdf]

Supplementary Table S1. Search Strategies from all databases

Search strategy on PubMed

Medical Subject Headings (MeSH) terms and Emtree terms used to conduct the search in the databases.

pulmonary disease, chronic obstructive[MeSH Terms] OR "chronic obstructive pulmonary disease"[All Fields] OR "COPD"[All Fields]) AND (("law" OR "regulation" OR "legal" OR "legislation" or regulat or "policy" or "policies") OR (("Legislation as Topic"[Mesh]) OR "Legislation, Medical"[Mesh]))) AND (("telehealth" OR "tele-health" OR "biomedical device*" OR "digital health technolog*" OR "mhealth" OR "m-health") OR ((Biomedical Technology[MeSH Terms]) OR (telemedicine[MeSH Terms AND Qualitative Study OR Qualitative Research])))*

Search strategy on Web of Science

chronic obstructive pulmonary disease" OR COPD AND regulation OR law OR legal OR legislation or regulat or policy or policies AND telehealth OR "tele-health" OR "biomedical device" OR "digital health technology" OR mhealth OR "m-health" OR "Biomedical Technology" OR telemedicine AND "Qualitative* OR "Qualitative Research"*

Search strategy Scopus

chronic obstructive pulmonary disease" OR copd AND regulation OR law OR legal OR legislation OR regulat OR policy OR policies AND telehealth OR "tele-health" OR "biomedical device" OR "digital health technology" OR mhealth OR "m-health" OR "Biomedical Technology" OR telemedicine AND "Qualitative" OR "Research"*

Search strategy JSTOR

(((COPD) AND (legislation)) AND (telehealth)) AND ((qualitative))
