# Supplementary material for: Evaluating the Impact of Regulatory Guidelines on Market Adoption and Implementation of Telehealth for COPD Patients: A Systematic Literature Review
Source: Healthcare (Basel). 2025 Nov 11;13(22):2858. doi: 10.3390/healthcare13222858 (PMC12652534; doi:10.3390/healthcare13222858)
Supplement: Supplementary file 1 [file healthcare-13-02858-s001.zip › Supplementary Table S2.pdf]

Supplementary Table S2. Frequency and percentage of the barriers mentioned in the included studies (n=18).

Barriers	N	%	Quotations / Examples
**Governmental (leaders & regulators)**			
Resource constraints	9	50	Limited staff and technical resources restricted telehealth use. [1,2]
Financial deficits	8	44	Unclear reimbursement and sustainability plans limit implementation.[3,4]
Community services	4	22	Weak integration between hospital and community respiratory services.[5,6]
Shared vision	6	33	Lack of a unified vision among stakeholders for digital health adoption. [7,8]
**Organisational & industry (policymakers / admins / payers)**			
Communication	9	50	Insufficient communication between care levels and teams impedes. [5,9]
Policy implementation	7	39	Gap between national telehealth policy and practice. [5,10]
TH failing	5	28	Telemonitoring enters a 'trough of disillusionment' when expectations outpace results. [11]
TH duration	4	22	Projects often fail to sustain beyond initial funding phase. [1,4]
Customization	5	28	mHealth features must be tailored to patients' unique needs and contexts. [6,12]
Technology complexity	10	56	Technical issues, low interoperability and usability concerns common. [13,14]
**Users (HCPs & patients)**			
Resistance to change	6	33	Frontline staff feared loss of autonomy with telehealth integration. [13]
Incomplete data	3	17	Weak data management and inconsistent evidence base. [11]
Data security	3	17	Concerns about confidentiality and data sharing between systems. [10,14]
Adoption	11	61	Adoption depends on perceived value and trust in the system. [9,15]
Workload	6	33	Remote monitoring increased documentation and follow-up work. [3,13]
Language	3	17	Language and health literacy barriers limited telehomecare effectiveness. [1,6]

Footnote: Date reported as frequency and percentage.

References

.1 Hunting, G.; Shahid, N.; Sahakyan, Y.; Fan, I.; Moneypenny, C.R.; Stanimirovic, A.; North, T.; Petrosyan, Y.; Krahn, M.D.; Rac, V.E. A multi-level qualitative analysis of Telehomecare in Ontario: challenges and opportunities. *BMC health services research* **2015**, *15*, 1-15.

.2 Slevin, P.; Kessie, T.; Cullen, J.; Butler, M.; Donnelly, S.; Caulfield, B. Exploring the barriers and facilitators for the use of digital health

technologies for the management of COPD: a qualitative study of clinician perceptions .*QJM: An International Journal of Medicine* **2020**, *113*, 163-172.
.3 Dirven, J.A.; Moser, A.; Tange, H.J.; Muris, J.W.; Van Schayck, O.C. An innovative COPD early detection programme in general practice: evaluating barriers to implementation. *npj Primary Care Respiratory Medicine* **2014**, *24*, 1-2.
.4 Walters, B.H.; Adams, S.A.; Nieboer, A.P.; Bal, R. Disease management projects and the Chronic Care Model in action: baseline qualitative research. *BMC Health Services Research* **2012**, *12*, 1-11.
.5 Hamilton, S.; Huby ,G.; Tierney, A.; Powell, A.; Kielmann, T.; Sheikh, A.; Pinnock, H. Mind the gap between policy imperatives and service provision: a qualitative study of the process of respiratory service development in England and Wales. *BMC Health Services Research* **200**.11-1 ,8 ,8
.6 Yadav, U.N.; Lloyd, J.; Baral, K.P.; Bhatta, N.; Mehata, S.; Harris, M. Evaluating the feasibility and acceptability of a co-design approach to developing an integrated model of care for people with multi-morbid COPD in rural Nepal: a qualitative study. *BMJ open* **2021**, *11*, e045175.
.7 An, Q.; Kelley, M.M.; Yen, P.-Y. Stakeholder mapping on the development of digital health interventions for self-management among patients with chronic obstructive pulmonary disease in China. *Studies in Health Technology and Informatics* **2022**, 1106-1107.
.8 Odeh, B.; Kayyali, R.; Nabhani-Gebara, S.; Philip, N. Implementing a telehealth service: nurses' perceptions and experiences. *British Journal of Nursing* **2014**, *23*, 1133-1137.
.9 van Lieshout, F.; Yang, R.; Stamenova, V.; Agarwal, P.; Cornejo Palma, D.; Sidhu, A.; Engel, K.; Erwood, A.; Bhatia, R.S.; Bhattacharyya, O. Evaluating the implementation of a Remote-Monitoring program for chronic obstructive pulmonary disease: qualitative methods from a service design perspective. *J Med Internet Res* **2020**, *22*, e18148.
.10 Rojahn, K.; Laplante, S.; Sloand, J.; Main, C.; Ibrahim, A.; Wild, J.; Sturt, N.; Areteou, T.; Johnson, K.I. Remote monitoring of chronic diseases: a landscape assessment of policies in four European countries. *PloS one* **2016**, *11*, e0155738.
.11 Elwyn, G.; Hardisty, A.R.; Peirce, S.C.; May, C.; Evans, R.; Robinson, D.K.; Bolton, C.E.; Yousef, Z.; Conley, E.C.; Rana, O.F. Detecting deterioration in patients with chronic disease using telemonitoring: navigating the 'trough of disillusionment'. *Journal of evaluation in clinical practice* **2012**, *18*, 896-903.
.12 Alwashmi, M.F.; Fitzpatrick, B.; Davis, E.; Farrell, J.; Gamble, J.-M.; Hawboldt, J. Features of a mobile health intervention to manage chronic obstructive pulmonary disease: a qualitative study. *Therapeutic Advances in Respiratory Disease* **2020**, *14*, 1753466620951044.
.13 Taylor, J.; Coates, E.; Brewster, L.; Mountain, G.; Wessels, B.; Hawley, M.S. Examining the use of telehealth in community nursing :identifying the factors affecting frontline staff acceptance and telehealth adoption. *Journal of advanced nursing* **2015**, *71*, 326-337.
.14 Jiang, Y.; Sun, P.; Chen, Z.; Guo, J.; Wang, S.; Liu, F.; Li, J. Patients' and healthcare providers' perceptions and experiences of telehealth use and online health information use in chronic disease management for older

patients with chronic obstructive pulmonary disease: a qualitative study. *BMC geriatrics* **2022**, *22*, 1-16.
.15 Gaveikaite, V.; Grundstrom, C.; Lourida, K ;.Winter, S.; Priori, R.; Chouvarda, I.; Maglaveras, N. Developing a strategic understanding of telehealth service adoption for COPD care management: A causal loop analysis of healthcare professionals. *PLoS One* **2020**, *15*, e0229619.
