# Supplementary material for: Evaluating the Impact of Regulatory Guidelines on Market Adoption and Implementation of Telehealth for COPD Patients: A Systematic Literature Review
Source: Healthcare (Basel). 2025 Nov 11;13(22):2858. doi: 10.3390/healthcare13222858 (PMC12652534; doi:10.3390/healthcare13222858)
Supplement: Supplementary file 1 [file healthcare-13-02858-s001.zip › Supplementary Table S3.pdf]

Supplementary Table S3. Frequency and percentage of the facilitators mentioned in the included studies

Facilitators	N	%	Quotation / Example
**Governmental (leaders & regulators)**			
Funding	11	39	Targeted grants and pilot funds supported early telehealth expansion. [1]
Investments	9	32	Investment in tele-infrastructure and ICT staff pivotal for success.[2]
Prioritise critical patients.	7	25	Telehealth directed to frequent exacerbators to reduce readmission risk.[3]
Universal vision for health system.	6	21	Shared policy vision for integrated chronic care guided implementation.[4]
**Organizational & industry (policymakers/admins/payers)**			
Communication platforms	12	43	Interactive apps and shared EHRs enhanced communication.[5]
Clear guidelines	10	36	Guidelines standardised referrals and reduced provider uncertainty. [6]
Training on TH	13	46	Staff training improved confidence in remote monitoring systems. [7]
Discharge plans from TH	6	21	Structured discharge planning ensured continuity of care. [8]
Feasible TH devices	11	39	Portable, affordable devices key for rural deployment.[9]
User-friendly TH devices	14	50	Simple interfaces and patient-centred design boosted adherence.[10]
**Users (HCPs & patients)**			
User engagement	14	50	Community co-design approaches enhanced local acceptance.[11]
Provide training on TH	12	43	Continuous coaching and refresher sessions supported sustained use. [7]
Data-sharing protocol	9	32	Formal data-sharing agreements-built trust among providers.[1]
Clear referral criteria	7	25	Defined eligibility criteria reduced inappropriate enrolments.[6]
Automated TH systems	8	29	Automation enabled real-time alerts for deterioration.[12]
Offer multilingual support	5	18	Translating apps and interfaces improved patient understanding.[8]

Footnote: Date reported as frequencies and percentages.

References

1. Rojahn, K.; Laplante, S.; Sloand, J.; Main, C.; Ibrahim, A.; Wild, J.; Sturt, N.; Areteou, T.; Johnson, K.I. Remote monitoring of chronic diseases: a landscape assessment of policies in four European countries. *PloS one* **2016**, *11*, e0155738.
2. Meiwald, A.; Gara-Adams, R.; Rowlandson, A.; Ma, Y.; Watz, H.; Ichinose, M.; Scullion, J.; Wilkinson, T.; Bhutani, M.; Weston, G. Qualitative validation of COPD evidenced care pathways in Japan, Canada, England, and Germany: common barriers to optimal COPD care. *International journal of chronic obstructive pulmonary disease* **2022**, 1507-1521.
3. Dirven, J.A.; Moser, A.; Tange, H.J.; Muris, J.W.; Van Schayck, O.C. An innovative COPD early detection programme in general practice: evaluating barriers to implementation. *npj Primary Care Respiratory Medicine* **2014**, *24*, 1-2.
4. Hamilton, S.; Huby, G.; Tierney, A.; Powell, A.; Kielmann, T.; Sheikh, A.; Pinnock, H. Mind the gap between policy imperatives and service provision: a qualitative study of the process of respiratory service development in England and Wales. *BMC Health Services Research* **2008**, *8*, 1-11.
5. Alwashmi, M.F.; Fitzpatrick, B.; Davis, E.; Farrell, J.; Gamble, J.-M.; Hawboldt, J. Features of a mobile health intervention to manage chronic obstructive pulmonary disease: a qualitative study. *Therapeutic Advances in Respiratory Disease* **2020**, *14*, 1753466620951044.
6. Odeh, B.; Kayyali, R.; Nabhani-Gebara, S.; Philip, N. Implementing a telehealth service: nurses' perceptions and experiences. *British Journal of Nursing* **2014**, *23*, 1133-1137.
7. Slevin, P.; Kessie, T.; Cullen, J.; Butler, M.; Donnelly, S.; Caulfield, B. Exploring the barriers and facilitators for the use of digital health technologies for the management of COPD: a qualitative study of clinician perceptions. *QJM: An International Journal of Medicine* **2020**, *113*, 163-172.
8. Hunting, G.; Shahid, N.; Sahakyan, Y.; Fan, I.; Moneypenny, C.R.; Stanimirovic, A.; North, T.; Petrosyan, Y.; Krahn, M.D.; Rac, V.E. A multi-level qualitative analysis of Telehomecare in Ontario: challenges and opportunities. *BMC health services research* **2015**, *15*, 1-15.
9. An, Q.; Kelley, M.M.; Yen, P.-Y. Stakeholder mapping on the development of digital health interventions for self-management among patients with chronic obstructive pulmonary disease in China. *Studies in Health Technology and Informatics* **2022**, 1106-1107.
10. Jiang, Y.; Sun, P.; Chen, Z.; Guo, J.; Wang, S.; Liu, F.; Li, J. Patients' and healthcare providers' perceptions and experiences of telehealth use and online health information use in chronic disease management for older patients with chronic obstructive pulmonary disease: a qualitative study. *BMC geriatrics* **2022**, *22*, 1-16.
11. Yadav, U.N.; Lloyd, J.; Baral, K.P.; Bhatta, N.; Mehata, S.; Harris, M. Evaluating the feasibility and acceptability of a co-design approach to developing an integrated model of care for people with multi-morbid COPD in rural Nepal: a qualitative study. *BMJ open* **2021**, *11*, e045175.
12. Elwyn, G.; Hardisty, A.R.; Peirce, S.C.; May, C.; Evans, R.; Robinson, D.K.; Bolton, C.E.; Yousef, Z.; Conley, E.C.; Rana, O.F. Detecting deterioration in

patients with chronic disease using telemonitoring: navigating the ‘trough of disillusionment’. *Journal of evaluation in clinical practice* **2012**, *18*, 896-903.
