# Supplementary material for: Evaluating the Impact of Regulatory Guidelines on Market Adoption and Implementation of Telehealth for COPD Patients: A Systematic Literature Review
Source: Healthcare (Basel). 2025 Nov 11;13(22):2858. doi: 10.3390/healthcare13222858 (PMC12652534; doi:10.3390/healthcare13222858)
Supplement: Supplementary file 1 [file healthcare-13-02858-s001.zip › Supplementary Table S4.pdf]

Supplementary Table S4. Comparative thematic analysis of barriers between High-income and Low-Income health systems.

Barrier Theme	High-Income Systems (HICs)	Low Income Systems (LICs)	Comparative Insight
Policy / Governance	Policy–practice gap; fragmented reimbursement and inconsistent intersectoral coordination.[1,2]	Absent or weak TH policies; poor infrastructure funding continuity and weak regulatory frameworks.[3,4]	HICs face policy misalignment. LICs face policy absence and infrastructure deficits.
Resources / Workforce	Time limitations, staff buy-in challenges, lack of workflow integration, and unclear TH roles.[5,6]	Limited trained staff, dependency on funded pilot projects, lack of institutional sustainability. [3,4]	HICs: workload redistribution. LICs: workforce insufficiency.
Technology / Infrastructure	Data overload, interoperability gaps, inconsistent device reliability across care levels.[5,7]	Frequent equipment failures, poor maintenance capacity, unstable internet connectivity. [3,4]	HICs: data complexity. LICs: technological fragility.
Patient Factors	Low digital literacy among elderly COPD patients, limited motivation.[1,5]	Low health and eHealth literacy, language barriers, device cost, and lack of awareness. [3,4]	HICs: fast digital development. LICs: literacy, economic, and linguistic inequities.
Ethical / Privacy	Liability risks and ethical concerns regarding remote patient monitoring and/or data management.[5]	Weak privacy regulation and patient mistrust in data protection. [3,4]	HICs: over-regulation and liability anxiety. LICs: No regulations and low trust.

References

1. Taylor, J.; Coates, E.; Brewster, L.; Mountain, G.; Wessels, B.; Hawley, M.S. Examining the use of telehealth in community nursing: identifying the factors affecting frontline staff acceptance and telehealth adoption. *Journal of advanced nursing* **2015**, *71*, 326-337.

2. Meiwald, A.; Gara-Adams, R.; Rowlandson, A.; Ma, Y.; Watz, H.; Ichinose, M.; Scullion, J.; Wilkinson, T.; Bhutani, M.; Weston, G. Qualitative validation of COPD evidenced care pathways in Japan, Canada, England, and Germany: common barriers to optimal COPD care. *International journal of chronic obstructive pulmonary disease* **2022**, 1507-1521.
3. Jiang, Y.; Sun, P.; Chen, Z.; Guo, J.; Wang, S.; Liu, F.; Li, J. Patients' and healthcare providers' perceptions and experiences of telehealth use and online health information use in chronic disease management for older patients with chronic obstructive pulmonary disease: a qualitative study. *BMC geriatrics* **2022**, *22*, 1-16.
4. Yadav, U.N.; Lloyd, J.; Baral, K.P.; Bhatta, N.; Mehata, S.; Harris, M. Evaluating the feasibility and acceptability of a co-design approach to developing an integrated model of care for people with multi-morbid COPD in rural Nepal: a qualitative study. *BMJ open* **2021**, *11*, e045175.
5. Slevin, P.; Kessie, T.; Cullen, J.; Butler, M.; Donnelly, S.; Caulfield, B. Exploring the barriers and facilitators for the use of digital health technologies for the management of COPD: a qualitative study of clinician perceptions. *QJM: An International Journal of Medicine* **2020**, *113*, 163-172.
6. Hunting, G.; Shahid, N.; Sahakyan, Y.; Fan, I.; Moneypenny, C.R.; Stanimirovic, A.; North, T.; Petrosyan, Y.; Krahn, M.D.; Rac, V.E. A multi-level qualitative analysis of Telehomecare in Ontario: challenges and opportunities. *BMC health services research* **2015**, *15*, 1-15.
7. Gaveikaite, V.; Grundstrom, C.; Lourida, K.; Winter, S.; Priori, R.; Chouvarda, I.; Maglaveras, N. Developing a strategic understanding of telehealth service adoption for COPD care management: A causal loop analysis of healthcare professionals. *PLoS One* **2020**, *15*, e0229619.
