# Supplementary material for: Evaluating the Impact of Regulatory Guidelines on Market Adoption and Implementation of Telehealth for COPD Patients: A Systematic Literature Review
Source: Healthcare (Basel). 2025 Nov 11;13(22):2858. doi: 10.3390/healthcare13222858 (PMC12652534; doi:10.3390/healthcare13222858)
Supplement: Supplementary file 1 [file healthcare-13-02858-s001.zip › Supplementary Table S5.pdf]

Supplementary Table S5. Comparative thematic analysis of facilitators between High-income and Low-Income health systems.

Facilitator Theme	High-Income Systems (HICs)	Low Income Systems (LICs)	Comparative Insight
Policy / Governance	Strong national digital strategies but implementation gaps; success when policy aligns with service delivery. [1,2]	Government co-design and alignment with primary health care strengthen feasibility. [3]	HICs require policy coherence. LICs require participatory governance and cross-sector partnerships.
Financing / Investment	Reimbursement incentives and innovation funds accelerate adoption; need long-term funding models.[1,2]	External or donor funding catalyzes pilots; local investment in ICT infrastructure essential for scalability.[4]	HICs: sustainability of funding. LICs: seed funding and infrastructure.
Technology / Infrastructure	Emphasis on reliable systems and integration with electronic medical records.[5,6]	Focus on affordable, user-friendly, and offline devices adapted to resource constraints. [4,7]	HICs: integration and automation.<br>LICs: affordability and accessibility.
Workforce / Training	Structured training and continuous professional development foster acceptance.[2]	Community-based training build local capacity.[3]	HICs: champion and leadership. LICs: training and local empowerment.
Communication / Data Systems	Data-sharing protocols and interoperability standards enable coordination across providers. [8]	Low-cost communication platforms and multilingual interfaces.[4]	HICs: data governance.<br>LICs: simplicity and accessibility.
Patient & User Engagement	Patient empowerment through co-design and usability increases sustained engagement. [5]	Culturally adapted interventions improve adoption.[3]	HICs: maintain engagement. LICs: foster inclusion through cultural and linguistic adaptation.

References

.1 Rojahn, K.; Laplante, S.; Sloand, J.; Main, C.; Ibrahim, A.; Wild, J.; Sturt, N.; Areteou, T.; Johnson, K.I. Remote monitoring of chronic diseases: a landscape assessment of policies in four European countries. *PloS one* **2016**, *11*, e0155738.

.2 Taylor, J ;.Coates, E.; Brewster, L.; Mountain, G.; Wessels, B.; Hawley, M.S. Examining the use of telehealth in community nursing: identifying the factors affecting frontline staff acceptance and telehealth adoption. *Journal of advanced nursing* **2015**, *71*, 326-337.

.3 Yadav, U.N.; Lloyd, J.; Baral, K.P.; Bhatta, N.; Mehata, S.; Harris, M. Evaluating the feasibility and acceptability of a co-design approach to developing an integrated model of care for people with multi-morbid COPD in rural Nepal: a qualitative study. *BMJ open* **2021**, *11*, e045175.

.4 Jiang, Y.; Sun, P.; Chen, Z.; Guo, J.; Wang, S.; Liu, F.; Li, J. Patients' and healthcare providers' perceptions and experiences of telehealth use and online health information use in chronic disease management for older patients with chronic obstructive pulmonary disease: a qualitative study. *BMC geriatrics* **2022**, *22*, 1-16.

.5 Slevin, P.; Kessie, T.; Cullen, J.; Butler, M.; Donnelly, S.; Caulfield, B. Exploring the barriers and facilitators for the use of digital health technologies for the management of COPD: a qualitative study of clinician perceptions. *QJM: An International Journal of Medicine* **2020**, *113*, 163-172.

.6 van Lieshout, F.; Yang, R.; Stamenova, V.; Agarwal, P.; Cornejo Palma, D.; Sidhu, A.; Engel, K.; Erwood ,A.; Bhatia, R.S.; Bhattacharyya, O. Evaluating the implementation of a Remote-Monitoring program for chronic obstructive pulmonary disease: qualitative methods from a service design perspective. *J Med Internet Res* **2020**, *22*, e18148.

.7 An, Q.; Kelley, M.M ;.Yen, P.-Y. Stakeholder mapping on the development of digital health interventions for self-management among patients with chronic obstructive pulmonary disease in China. *Studies in Health Technology and Informatics* **2022**, 1106-1107.

.8 Gaveikaite, V.; Grundstrom, C.; Lourida, K.; Winter, S.; Priori, R.; Chouvarda, I.; Maglaveras, N. Developing a strategic understanding of telehealth service adoption for COPD care management: A causal loop analysis of healthcare professionals. *PLoS One* **2020**, *15*, e0229619.
