# Supplementary material for: Evaluating the Impact of Regulatory Guidelines on Market Adoption and Implementation of Telehealth for COPD Patients: A Systematic Literature Review
Source: Healthcare (Basel). 2025 Nov 11;13(22):2858. doi: 10.3390/healthcare13222858 (PMC12652534; doi:10.3390/healthcare13222858)
Supplement: Supplementary file 1 [file healthcare-13-02858-s001.zip › Supplementary Table S6.pdf]

Supplementary Table S6. Critical appraisal of grey literature based on adopted tools from the AACODS checklist (Authority, Accuracy, Coverage, Objectivity, Date, Significance). [1]

Document/Report	Accuracy	Authority	Coverage	Objectivity	Date	Significance	AACODS score
WHO(Global) [2]	Yes	Yes	Yes	Yes	Yes	Yes	******
WHO(Global) [3]	Yes	Yes	Yes	Yes	Yes	Yes	******
WHO(Global) [4]	Yes	Yes	Yes	Yes	Yes	Yes	******
WHO (Global) [5]	Yes	Yes	Yes	Yes	Yes	Yes	******
UK (NHS) [6]	Yes	Yes	Yes	Yes	Yes	Yes	******
USA (CIH) [7]	Yes	Yes	Yes	Yes	Yes	Yes	******
Saudi Arabia (MoH) [8]	Yes	Yes	Yes	Yes	Yes	Yes	******
Saudi Arabia(CHI)[9]	Yes	Yes	Yes	Yes	Yes	Yes	******
Saudi Arabia (MoH)[10]	Yes	Yes	Yes	Yes	Yes	Yes	******
Saudi Arabia (SFDA) [11]	Yes	Yes	Yes	Yes	Yes	Yes	******
Saudi Arabia (SFDA)[12]	Yes	Yes	Yes	Yes	Yes	Yes	******
Indonesia (MoH)[13]	Yes	Yes	Yes	Yes	Yes	Yes	******
Thailand (MoPH)[13]	Yes	Yes	Yes	Yes	Yes	Yes	******
Vietnam (MoH)[13]	Yes	Yes	Yes	Yes	Yes	Yes	******
Singapore (MoH)[14]	Yes	Yes	Yes	Yes	Yes	Yes	******
Indian (MCI)[9]	Yes	Yes	Yes	Yes	Yes	Yes	******
Brazil (Review paper) [15]	Yes	No	Yes	Yes	Yes	Yes	*****

Footnote: WHO: World health Organization, NHS; National Health Services, USA; United States, CIH; Cicero Institute for health, MoH; Ministry of Health, CHI; Council Health Insurance, SFDA; Food and drug authority, MoPH; Ministry of Public Health, MCI; Medical Council of India

AACODS score: ****** possible score can be achieved based on Accuracy, Authority, Coverage, Objectivity, Date, and Significance.

References

1. Tyndall, J. AACODS checklist. Available online: http://dspace.flinders.edu.au/dspace/ (accessed on 22 October 2025).
2. WHO. *National eHealth Strategy Toolkit*; World Health Organization: 2012.
3. WHO. *WHO-ITU global standard for accessibility of telehealth services*; World Health Organization: 2020.
4. WHO. *Recommendations on digital interventions for health system strengthening*; World Health Organization: 2022.
5. WHO. *Digital Implementation Investment Guide (DIIG)*; World Health Organization: 2012.
6. Alison Dennis, S.N. *Issues with regulation of telemedicine in the UK.*; TaylorWessing 2022.
7. Ally Perkins Josh Archambault , V.N. *State Policy Agenda for Telehealth Innovation*; 2024.
8. MoH. *Legal Regulations for Telehealth Services* Ministry of Health: 2022.
9. MCI. *Telemedicine Practice Guidelines Enabling Registered Medical Practitioners to Provide Healthcare Using Telemedicine*; Medical Council of India, 2023.
10. MoH. *The governing rules of Telehealth in the kingdom of Saudi Arabia*; Ministry of Health: 2021.
11. SFDA. *Requirements for approving telehealth devices in clinical settings*; Saudi Food and Drug Authority, 2021.
12. SFDA. *Reguirments for telehaelth devices martking*; Saudi Food and Drug Authority: 2023.
13. Intan Sabrina, M.; Defi, I.R. Telemedicine guidelines in South East Asia—a scoping review. *Frontiers in neurology* **2021**, *11*, 581649.
14. HSA. *Regulatory guideline for telehealth products*; The Health Sciences Authority: 2021; p. 24.
15. Silva, A.B.; da Silva, R.M.; Ribeiro, G.d.R.; Guedes, A.C.C.M.; Santos, D.L.; Nepomuceno, C.C.; Caetano, R. Three decades of telemedicine in Brazil: Mapping the regulatory framework from 1990 to 2018. *PLoS one* **2020**, *15*, e0242869.
